# Supplementary material for: Multicountry genomic analysis underscores regional cholera spread in Africa
Source: Nat Commun. 2026 Feb 9;17:2539. doi: 10.1038/s41467-026-68642-7 (PMC13000271; doi:10.1038/s41467-026-68642-7)
Supplement: Supplementary file 1 — Supplementary Information [file 41467_2026_68642_MOESM1_ESM.pdf]

Multicountry genomic analysis underscores regional cholera spread in Africa

SUPPLEMENTARY NOTE 1

CholGEN Consortium Authors

Catholic University of Bukavu. Patrick Musole Bugeme

CDC Malawi. Elizabeth Kampira

Epicentre. Flavio Finger, Rachel Mahamba

Institut National de Recherche Biomédicale (DRC). Joel Kakwanda Kanyama, Emmanuel Lofiko Lokilo, Pauline-Chloé Kayembe Muswamba, Princesse Paku Tshambu

London School of Hygiene and Tropical Medicine. Chloe Hutchins, Jackie Knee

National Health Laboratory & Diagnostic Services (NHLDS/CPHL), Ministry of Health (Uganda): Grace Alenyo, Lydia Bulyaba, Benedict Kanamwanji, Stephen Kanyerezi, Moses Murungi, Ritah Namusoosa, Hellen Rosette Oundo, Joseph Sekate, William Senyonga, Julius Sseruyange, Godwin Tusabe, Tenywa Wilson

National Laboratory of Lubumbashi (DRC): Jacques Muzinga

National Program of Elimination of cholera, Ministry of Health (DRC): Doudou Boloweti Batumbo

National Public Health Laboratory, Ministry of Public Health (Cameroon): Yves Amang, Chanceline Bilounga Ndongo, Yvette Ebogo, Elodie Edinga, Linda Esso, Francoise Fouda, Etienne Guenou, Clarisse Kila, Nadia Jacqueline Mandeng, Moise Christian Junior Meka, Marie Claire Okomo Assoumou, Yvette Wirba, Sylvain Engamba

Nigeria Centre for Disease Control and Prevention: Sophiyah Damilola Adelakun, Osaoghomwen Amiebenomo, James Avong, Deborah Effiong, Eme Ekeng, Kenneth Chukwuemeka Ikeata, Khadijah Imam, Ikechukwu Nnaji, Zayyanatu Nuru, Richard Olulowo Ojedele, Deborah Okomayin, Chidiebere Opara, Victor Oripenaiye

Public Health Institute of Malawi: Arthur Baluwa, Ephrone Keddie Banda, Chifundo Banda, Mphatso Katumbule Bukhu, Yollam Chavula, Moses Chitenje, Michael Hauli, Patrick Kalengo, Mphatso Kanjiru, Grace Kusakara, Innocent Malolo, Happy Manda, Christopher Misomali, Annie Mwale, Andrew Mzumara, Bright Odala, Louis Panja

Rodolphe Merieux Institut National de Recherche Biomédicale-Goma (DRC): Tavia Matamu Bodisa, Jules Namugusha Cizungu, Yves Birindwa Hamisi, Pascal Nzoloka Kabuyaya, Adèle Kamaliro Kavira, Faida Kitoga, Brigitte Modrada Madakpa, Jeriel Mufungizi, Michel Ngimba, Zéphanie Kalimuli Paluku, Espérance Tsilabia Tsiwedi

Zambia Cholera Task Force & Centre for Infectious Disease Research Zambia: Caroline C. Chisenga, Michelo Simuyandi

Zambia National Public Health Institute: Priscilla Nkonde Gardner, Muzala Kapin'a, Peter Chibale Mwansa, Nchimunya Siabeenzu

SUPPLEMENTARY FIGURES

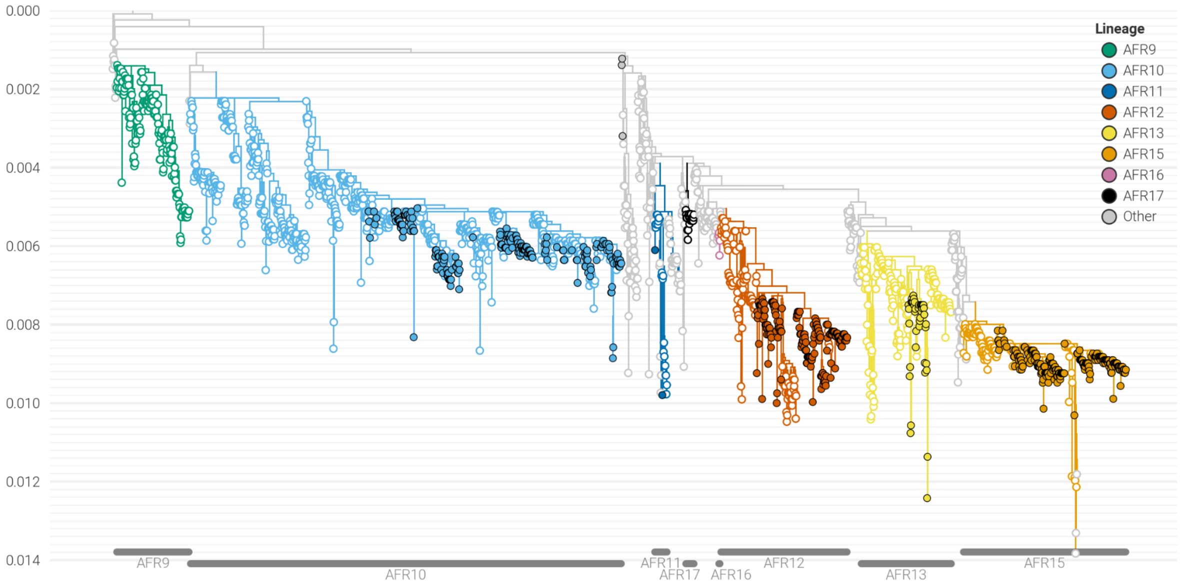

Supplementary Figure 1. Evolutionary history of the third wave of 7PET. Maximum-likelihood phylogeny with taxa labeled by the assigned lineage. Only lineages known to circulate in sub-Saharan Africa are colored. Filled circles: genomes generated by CholGEN Member States. Open circles: previously published genomes.

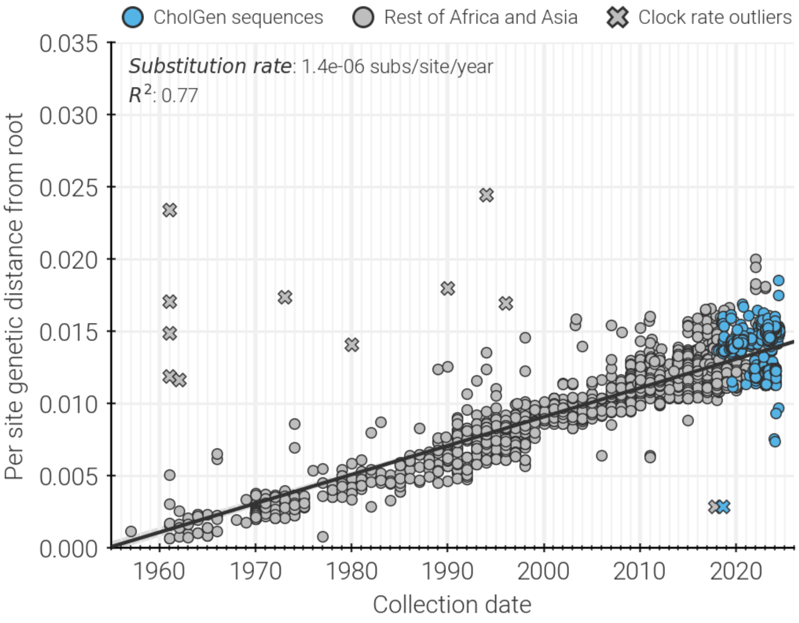

Supplementary Figure 2. Regression of root-to-tip genetic distances against sampling dates, for sequences belonging to the 7th pandemic clone of cholera. Sequences in gray represent those previously published, while sequences in blue represent those generated by CholGEN. Sequences indicated by a cross mark are more than three interquartile distances from the regression and have been removed from BEAST analyses.

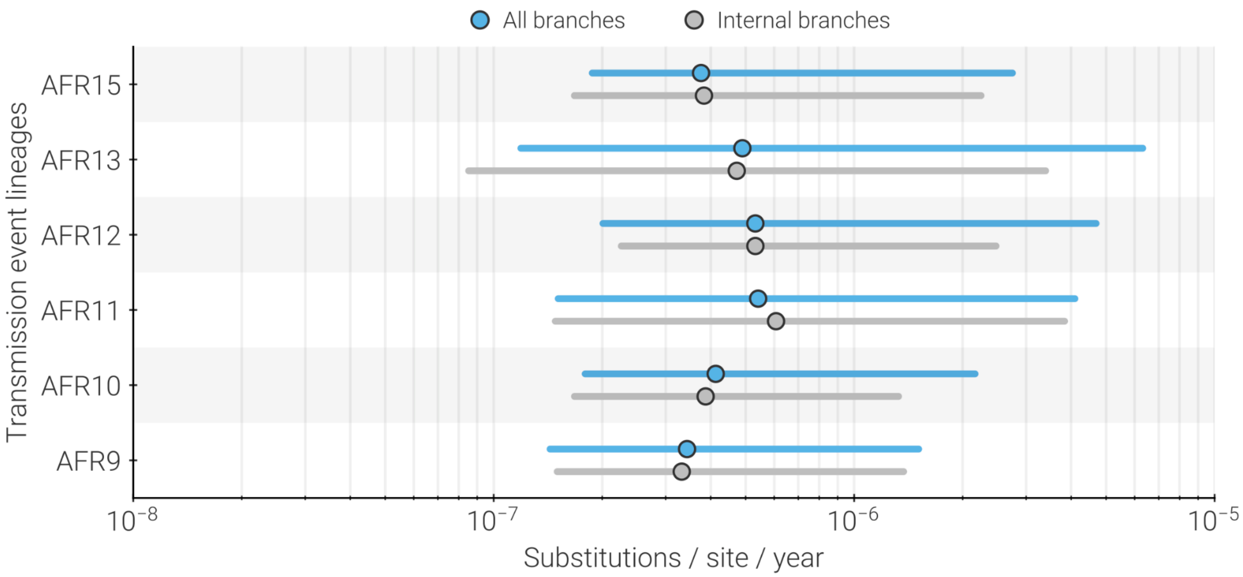

Supplementary Figure 3. Distribution of substitution rates found on branches belonging to each of the currently circulating lineages. For each lineage, the range of substitution rates estimated for each branch is shown. Dot indicates median value while bar indicates 95% HPD. Blue represents the range of all branches, while gray indicates internal branches only. Note that the x-axis is log-scaled.

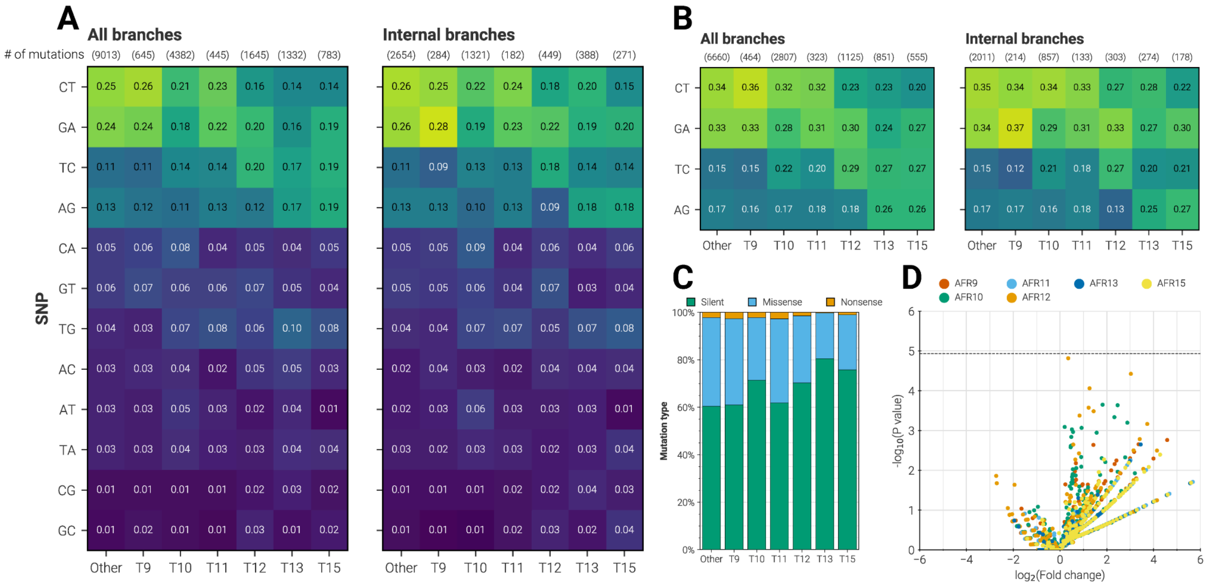

Supplementary Figure 4. Mutation profile of African lineages. (**A**) Proportion of single-nucleotide mutations by specific mutation type, on all versus internal-only branches. Pearson's chi-squared test indicates no significant differences between any lineage and non-lineage branches ("Other"). (**B**) Same as **A**, but only transitions are counted. No significant differences observed. (**C**) Proportion of silent, missense, and nonsense mutations occurring on branches of each lineage. (**D**) Non-synonymous mutations were assigned to Gene Ontology biological processes. Scatter plot displays the statistical over-enrichment of biological processes mutated on each lineage's branches as determined with a Fisher's Exact test (null hypothesis is random distribution of mutations). No biological processes for any lineage had a P value less than the Bonferroni-corrected significance level (dashed line).

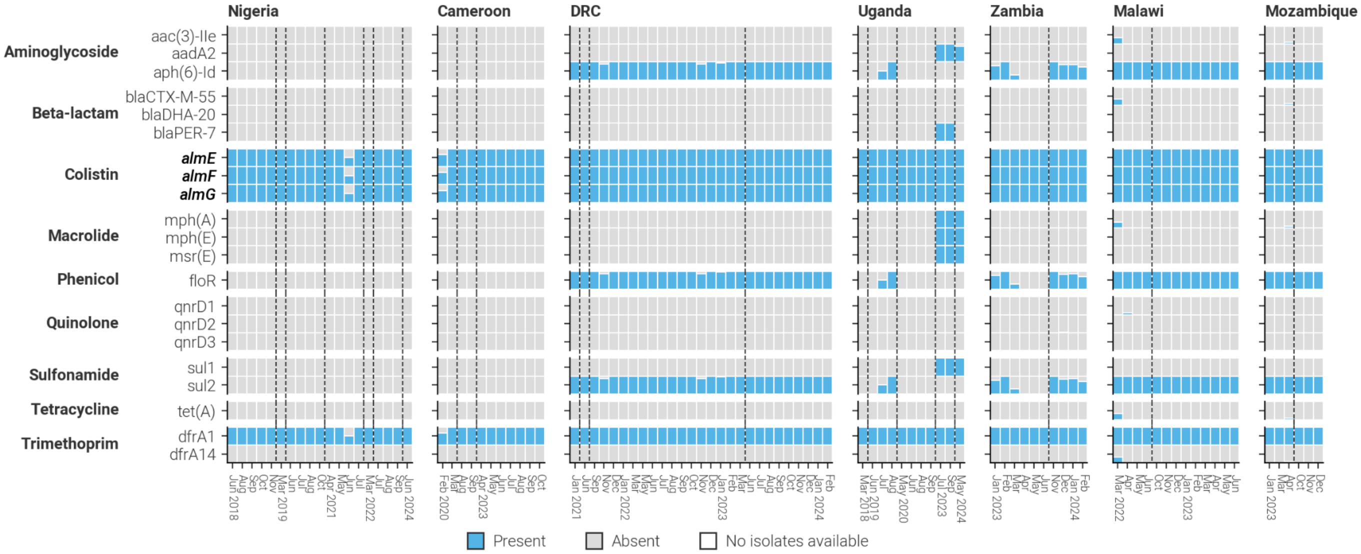

Supplementary Figure 5. Antimicrobial resistance profile of cholera in CholGEN Member states. The proportion of isolates collected in each month and country which carry specific antimicrobial resistance genes. Blue bars represent presence and gray represents absence of the gene. Countries are ordered generally West-to-East, and genes are organized by the class of antibiotic that they provide resistance to. Bolded genes are present on the two genomic chromosomes of cholera, whereas unbolded genes are likely present on mobile genetic elements. Dashed vertical lines indicate a break in the temporal x-axis.

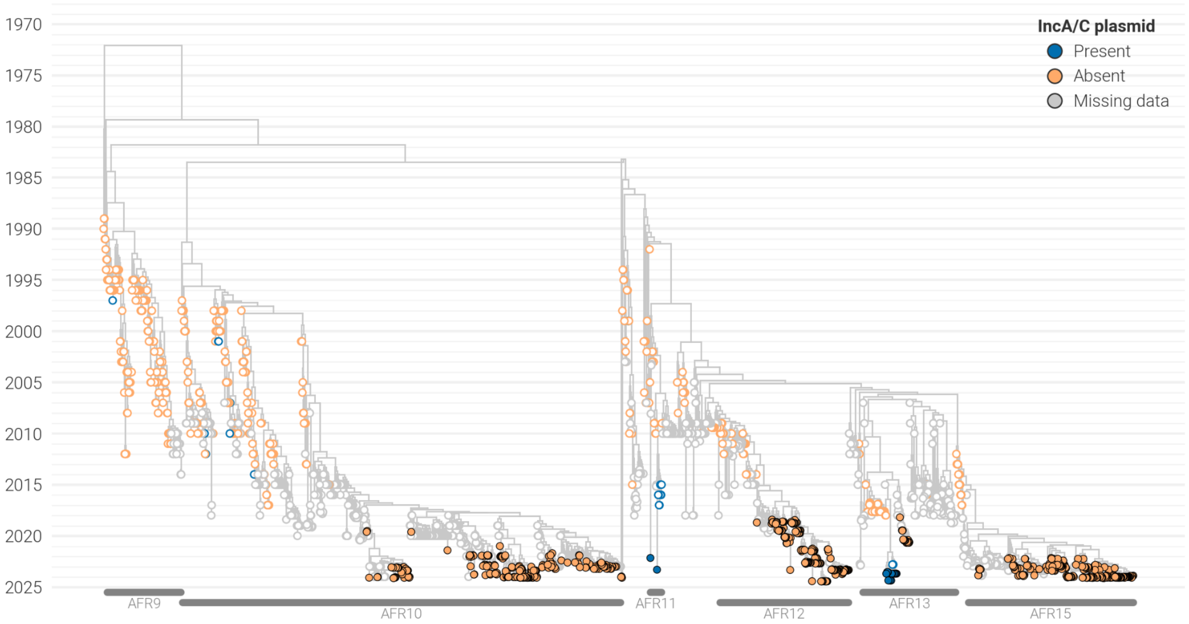

Supplementary Figure 6. Acquisition of the IncA/C plasmid amongst 7PET isolates. Maximum likelihood tree with taxa labeled by whether the IncA/C plasmid was detected or not. Filled circles: genomes generated by CholGEN Member States. Open circles: previously published genomes.
