# Supplementary material for: Multicountry genomic analysis underscores regional cholera spread in Africa
Source: Nat Commun. 2026 Feb 9;17:2539. doi: 10.1038/s41467-026-68642-7 (PMC13000271; doi:10.1038/s41467-026-68642-7)
Supplement: Supplementary file 2 — Description of Additional Supplementary Files [file 41467_2026_68642_MOESM2_ESM.pdf]

Description of Additional Supplementary Files

Supplementary Data 1. Details of V. cholerae O1 isolates generated by this study. Includes metadata, sequencing and assembly statistics, and antimicrobial resistance profiling data for all sequences included in analyses.

Supplementary Data 2. List of publicly available V. cholerae O1 isolates used in this study. Metadata including collection location, year, assigned lineage, and accession number for all sequences included in the background dataset for this study.
