# Supplementary material for: Multicountry genomic analysis underscores regional cholera spread in Africa
Source: Nat Commun. 2026 Feb 9;17:2539. doi: 10.1038/s41467-026-68642-7 (PMC13000271; doi:10.1038/s41467-026-68642-7)
Supplement: Supplementary file 5 — Reporting Summary [file 41467_2026_68642_MOESM5_ESM.pdf]

nature portfolio

Corresponding author(s): Shirlee Wohl
Sofonias Kifle Tessema

Last updated by author(s): Sep 20, 2024

Reporting Summary

Nature Portfolio wishes to improve the reproducibility of the work that we publish. This form provides structure for consistency and transparency in reporting. For further information on Nature Portfolio policies, see our Editorial Policies and the Editorial Policy Checklist.

Statistics

For all statistical analyses, confirm that the following items are present in the figure legend, table legend, main text, or Methods section.

n/a	Confirmed	
☐	☒	The exact sample size (*n*) for each experimental group/condition, given as a discrete number and unit of measurement
☐	☒	A statement on whether measurements were taken from distinct samples or whether the same sample was measured repeatedly
☐	☒	The statistical test(s) used AND whether they are one- or two-sided<br>*Only common tests should be described solely by name; describe more complex techniques in the Methods section.*
☐	☒	A description of all covariates tested
☒	☐	A description of any assumptions or corrections, such as tests of normality and adjustment for multiple comparisons
☐	☒	A full description of the statistical parameters including central tendency (e.g. means) or other basic estimates (e.g. regression coefficient) AND variation (e.g. standard deviation) or associated estimates of uncertainty (e.g. confidence intervals)
☐	☒	For null hypothesis testing, the test statistic (e.g. *F*, *t*, *r*) with confidence intervals, effect sizes, degrees of freedom and *P* value noted<br>*Give P values as exact values whenever suitable.*
☐	☒	For Bayesian analysis, information on the choice of priors and Markov chain Monte Carlo settings
☒	☐	For hierarchical and complex designs, identification of the appropriate level for tests and full reporting of outcomes
☐	☒	Estimates of effect sizes (e.g. Cohen's *d*, Pearson's *r*), indicating how they were calculated

Our web collection on statistics for biologists contains articles on many of the points above.

Software and code

Policy information about availability of computer code

Data collection	All data collection code is available in the Github repository for this manuscript (https://github.com/CholGen/RegionalAnalysis-2024). Our custom scripts and workflows use the following software: FastQC v0.12.0, bwa-mem v0.7.17, BCFTools v1.20, snippy v4.6.0, bacpage v2024.03.08, and the TheiaProk Illumina paired-end sequencing workflow v3.1.1.
Data analysis	All data analysis code is available in the Github repository for this manuscript (https://github.com/CholGen/RegionalAnalysis-2024). Along with custom python scripts, we also used the following tools: gubbins v2.3.4, SNP-sites v2.5.1, IQ-TREE2 v2.3.6, BEAST v1.10.5, Tracer v1.7.2, Beastiary v1.8.3, Treetime v0.11.3

For manuscripts utilizing custom algorithms or software that are central to the research but not yet described in published literature, software must be made available to editors and reviewers. We strongly encourage code deposition in a community repository (e.g. GitHub). See the Nature Portfolio guidelines for submitting code & software for further information.

Data

Policy information about availability of data

All manuscripts must include a data availability statement. This statement should provide the following information, where applicable:

- Accession codes, unique identifiers, or web links for publicly available datasets
- A description of any restrictions on data availability
- For clinical datasets or third party data, please ensure that the statement adheres to our policy

Raw sequencing reads are available on NCBI under the BioProject accession ID PRJNA1145341 and Sequence Read Archive accession IDs are provided in Supp. Table 1. Accession IDs for the publicly available sequences acquired from NCBI or VibrioWatch are provided in Supp. Table 2.

Research involving human participants, their data, or biological material

Policy information about studies with human participants or human data. See also policy information about sex, gender (identity/presentation), and sexual orientation and race, ethnicity and racism.

Reporting on sex and gender	Sex and gender data was not considered in this study.
Reporting on race, ethnicity, or other socially relevant groupings	No covariates pertaining to race, ethnicity, or other socially relevant groupings were used in this study.
Population characteristics	No population characteristics were used in this study.
Recruitment	All samples were collected as part of routine surveillance; individuals were not specifically recruited for this study.
Ethics oversight	In Cameroon, ethical approval was granted by the National Committee on Ethics in Research for Human Health (2024/02/1640/CE/CNERSH/SP). In DRC, ethical approval was granted by the Board of the Ethics Committee of the School of Public Health at the University of Kinshasa (ESP/CE/148/2023 and ESP/CE/149/2023). In Malawi, this work was approved by the National Health Sciences Research Committee (Protocol #867). In Mozambique, ethical approval was granted by the National Bioethics Committee for Health (335/CNBS/23). In Nigeria, ethical approval was not required as it is based on data from Nigeria's national surveillance program, collected by the Nigeria Centre for Disease Control. In Uganda, ethical approval was granted by the Uganda Ministry of Health National Health Laboratory Services (UNHL-2024-88). In Zambia, ethical approval was granted by the University of Zambia Biomedical Research Ethics Committee (UNZA-7540/2025).

Note that full information on the approval of the study protocol must also be provided in the manuscript.

Field-specific reporting

Please select the one below that is the best fit for your research. If you are not sure, read the appropriate sections before making your selection.

☐ Life sciences ☐ Behavioural & social sciences ☒ Ecological, evolutionary & environmental sciences

For a reference copy of the document with all sections, see nature.com/documents/nr-reporting-summary-flat.pdf

Ecological, evolutionary & environmental sciences study design

All studies must disclose on these points even when the disclosure is negative.

Study description	To examine recent cholera transmission patterns, including the rapid spread of the AFR15 lineage linked to unusually large outbreaks in Southern Africa, we generated genomic data from 768 Vibrio cholerae O1 isolates collected primarily between 2019 and 2024. Our analysis compares continental and regional transmission dynamics of V. cholerae across different African regions, explores genotypic and evolutionary distinctions between cholera strains, and assesses the diversity of cholera present within each CholGEN Member State.
Research sample	Our research sample consisted of 1,220 Vibrio cholerae isolates collected from clinically suspected cholera cases in Cameroon, DRC, Malawi, Mozambique, Nigeria, Uganda, and Zambia, collected between 2018 and 2024. Samples were collected as part of routine surveillance and therefore were not restricted to particular demographic groups (e.g., sex, age).
Sampling strategy	Vibrio cholerae Isolates came from randomly selected patients from cholera treatment centers during outbreaks or from endemic areas. Our sampling was retrospective and captured most, if not all available samples due to the historic undersampling of cholera in Africa. As a result, we did not perform sample size calculations, though we note that the resulting genomic dataset is the largest V. cholerae whole genome sequencing dataset that has been generated to date for most of the CholGEN member states.
Data collection	Stool samples were collected as part of routine surveillance following national standards for data collection in each CholGEN Member State. Stool samples detected as cholera positive with a Cholera Rapid Diagnostic Test were transported in Cary-Blair media to laboratories then cultured on Thiosulfate-citrate-bile salts-sucrose agar medium and incubated for 20–24 hours, before a single

	colony was picked and DNA extracted. Sequencing libraries were generated from the DNA using an Illumina DNA Prep Kit and sequenced on a MiSeq or NextSeq 2000 using 300-cycle kits.
Timing and spatial scale	Isolates were collected between 2018 and 2024 in Cameroon, DRC, Malawi, Mozambique, Nigeria, Uganda, and Zambia.
Data exclusions	Isolates with at least 90% of their reads mapped to the reference genome, had median coverage across all positions of the reference genome of at least 15, and had less than 10% ambiguous nucleotides were retained for the analysis. Of the 1220 isolates sequenced, 492 samples, representing 40.3% of all samples, did not meet these criteria and were excluded from the analysis.
Reproducibility	Negative controls were utilized during all stages of sample processing to assess potential cross-contamination. Samples indicated by cross-contamination were excluded from analysis. For maximum-likelihood phylogenetic analysis, branch support values were assessed by 1000 bootstraps. For bayesian phylogenetic analyses, two independent MCMC chains of 100 millions steps were run. Estimated model parameters were found to be consistent between the two chains.
Randomization	All sequences/samples that met our thresholds for quality were included in the study. Additionally, we did not perform experiments to evaluate the effects of specific groups or treatments. Therefore, randomization was not required.
Blinding	Blinding was not applicable to this study because we did not perform experiments to determine the effect of specifics groups or treatments.

Did the study involve field work? ☐ Yes ☒ No

Reporting for specific materials, systems and methods

We require information from authors about some types of materials, experimental systems and methods used in many studies. Here, indicate whether each material, system or method listed is relevant to your study. If you are not sure if a list item applies to your research, read the appropriate section before selecting a response.

Materials & experimental systems

n/a	Involved in the study
☒	☐ Antibodies
☒	☐ Eukaryotic cell lines
☒	☐ Palaeontology and archaeology
☒	☐ Animals and other organisms
☒	☐ Clinical data
☒	☐ Dual use research of concern
☒	☐ Plants

Methods

n/a	Involved in the study
☒	☐ ChIP-seq
☒	☐ Flow cytometry
☒	☐ MRI-based neuroimaging

Plants

Seed stocks	*Report on the source of all seed stocks or other plant material used. If applicable, state the seed stock centre and catalogue number. If plant specimens were collected from the field, describe the collection location, date and sampling procedures.*
Novel plant genotypes	*Describe the methods by which all novel plant genotypes were produced. This includes those generated by transgenic approaches, gene editing, chemical/radiation-based mutagenesis and hybridization. For transgenic lines, describe the transformation method, the number of independent lines analyzed and the generation upon which experiments were performed. For gene-edited lines, describe the editor used, the endogenous sequence targeted for editing, the targeting guide RNA sequence (if applicable) and how the editor was applied.*
Authentication	*Describe any authentication procedures for each seed stock used or novel genotype generated. Describe any experiments used to assess the effect of a mutation and, where applicable, how potential secondary effects (e.g. second site T-DNA insertions, mosiacism, off-target gene editing) were examined.*
